# Activity of Antioxidant Enzymes in the Tumor and Adjacent Noncancerous Tissues of Non-Small-Cell Lung Cancer

**DOI:** 10.1155/2019/2901840

**Published:** 2019-10-31

**Authors:** Marzena Zalewska-Ziob, Brygida Adamek, Janusz Kasperczyk, Ewa Romuk, Edyta Hudziec, Ewa Chwalińska, Katarzyna Dobija-Kubica, Paweł Rogoziński, Krzysztof Bruliński

**Affiliations:** ^1^Chair and Department of Medical and Molecular Biology, School of Medicine with the Division of Dentistry in Zabrze, Medical University of Silesia, Katowice, Poland; ^2^Department of Basic Medical Sciences, School of Public Health in Bytom, Medical University of Silesia, Katowice, Poland; ^3^Chair and Department of Environmental Medicine and Epidemiology, School of Medicine with the Division of Dentistry in Zabrze, Medical University of Silesia, Katowice, Poland; ^4^Department of Biochemistry, School of Medicine with the Division of Dentistry in Zabrze, Medical University of Silesia, Katowice, Poland; ^5^John Paul II Hospital of Oncology, General Surgery Ward, Bielsko Biała, Poland; ^6^Pulmonology and Thoracic Surgery Center in Bystra, Thoracic Surgery Ward, Bystra, Poland

## Abstract

Lung tissue is directly exposed to high oxygen pressure, as well as increased endogenous and exogenous oxidative stress. Reactive oxygen species (ROS) generated in these conditions play an important role in the initiation and promotion of neoplastic growth. In response to oxidative stress, the antioxidant activity increases and minimizes ROS-induced injury in experimental systems. The aim of the present study was to evaluate the activity of antioxidant enzymes, such as superoxide dismutase (SOD; isoforms: Cu/ZnSOD and MnSOD), catalase (CAT), glutathione peroxidase (GPx), glutathione reductase (GR), and glutathione S-transferase (GST), along with the concentration of malondialdehyde (MDA) in tumor and adjacent noncancerous tissues of two histological types of NSCLC, i.e., adenocarcinoma and squamous cell carcinoma, collected from 53 individuals with surgically resectable NSCLC. MDA concentration was similar in tumors compared with adjacent noncancerous tissues. Tumor cells had low MnSOD activity, usually low Cu/ZnSOD activity, and almost always low catalase activity compared with those of the corresponding tumor-free lung tissues. Activities of GSH-related enzymes were significantly higher in tumor tissues, irrespective of the histological type of cancer. This pattern of antioxidant enzymes activity could possibly be the way by which tumor cells protect themselves against increased oxidative stress.

## 1. Introduction

During the last ten decades, lung cancer has become one of the most frequently occurring cancers and it is the leading cause of cancer-related death worldwide [1, 2]. Lung cancer usually originates from the basal epithelial cells and is classified into two types, namely, non-small-cell lung cancer (NSCLC), accounting for approximately 85% of all the cases, and small-cell lung cancer (SCLC), accounting for the remaining 15% of the cases with NSCLC. Based on the histological features, NSCLCs are classified into adenocarcinoma, squamous cell carcinoma, and large cell carcinoma, accounting for 40%, 20%, and 3% of the total lung cancer cases, respectively [3, 4].

The lung is directly exposed to high oxygen pressure, environmental irritants, and pollutants including oxidants, such as oxidant gases, ultrafine particulate materials, nanoparticles from industrial pollution, and car exhaust fumes, and smoking, all of which generate free radicals. This results in oxidative stress in the lungs and other organs of the body. The inflammatory response mediated by the inhalation of microbes, mainly viruses and bacteria, is also known to be an additional endogenous source of oxidative stress [5, 6].

Reactive oxygen species (ROS) are an integral part of the cell's oxygen metabolism which play an important role in several cellular processes at physiological concentrations by activating signaling pathways necessary for cell growth and proliferation. However, an excessive production of ROS damages important macromolecules, such as DNA, proteins, and lipids [7–9]. Malondialdehyde (MDA), one of the end-products of lipid peroxidation, is a highly toxic compound, which oxidatively modifies the macromolecules within the cells by reacting with imino (=NH) and sulphydryl (-SH) groups of proteins and DNA. MDA is considered to be a biomarker of lipid oxidative damage, especially those incorporated into the cell membranes [10, 11].

The lungs are protected against these oxidants by a variety of mechanisms which include a complex system of antioxidant enzymes, namely superoxide dismutase (SOD), glutathione peroxidase (GPx), glutathione reductase (GR), catalase (CAT), and nonenzymatic antioxidants (e.g., glutathione (GSH); vitamins A, C, D, and E; and *β*-carotene) [5]. The destructive chain of reactions initiated by ROS can be prevented by antioxidant enzymes; however, the inability of antioxidant enzymes to counteract the intracellular ROS levels leads to metabolic disturbances and cell death.

The first line of defense against ROS is SOD, which catalyzes the dismutation of superoxide anion (O_2_^•−^) into O_2_ and hydrogen peroxide (H_2_O_2_). Three isoforms of SOD exist in mammals: the cytoplasmic Cu/ZnSOD (SOD1), the mitochondrial MnSOD (SOD2), and the extracellular SOD (ECSOD, SOD3), all of which require catalytic metal (Cu or Mn) for activation and have been detected in human lung tissues. H_2_O_2_ generated as a result of the dismutation of O_2_^•−^ by SOD is further reduced to H_2_O by CAT or GPx [5, 12–14].

GSH, a thiol-group containing tripeptide, is synthesized from three amino acids, namely glycine, cysteine, and glutamate. GSH confers protection against oxidative stress by reducing hydroperoxides, quenching free radicals, and detoxifying xenobiotics [15]. The liver is the primary site of total body GSH turnover and accounts for over 90% of the GSH inflow into the systemic circulation. However, the concentration of GSH in the epithelial lining of human lungs is ~140 times higher than that in the circulation [5, 16]. The GSH-dependent antioxidant system consists of GSH and GSH-related enzymes which include glutathione S-transferase (GST), GPx, and GR [17]. GST catalyzes the conjugation of GSH with a variety of toxic compounds, including oxidative intermediates (such as lipids and DNA hydroperoxides and aldehydes), thereby, rendering them less toxic and facilitating their removal from the cells [18, 19]. GPx catalyzes the reduction of hydroperoxides, including lipid hydroperoxides, to water and the corresponding stable alcohols by using GSH as a substrate. This results in the oxidation of GSH yielding glutathione disulfide (GSSG), which is converted back by GR to its reduced form (GSH) [8, 17]. The shifting of the GSH/GSSG ratio towards the oxidized state in response to various intra- and extracellular environmental conditions in turn activates several signaling pathways (including protein kinase B, protein phosphatases 1 and 2A, calcineurin, nuclear factor *κ*B, c-Jun N-terminal kinase, apoptosis signal-regulated kinase 1, and mitogen-activated protein kinase), which reduces cell proliferation and increases apoptosis [8, 20].

Although oxidative stress has been implicated in several diseases including cancer, the mechanisms responsible for the induction of ROS in cancerous cells have not been fully understood. It is known that inflammation, oncogenic signals, DNA mutations, and dysfunction in the respiratory chain play an important role in inducing oxidative stress [8, 9]. The present study aims at evaluating the activity of antioxidant enzymes, such as SOD (Cu/ZnSOD, and MnSOD), CAT, GPx, GR, and GST along with the concentration of MDA in tumor and adjacent noncancerous tissues of two histological types of NSCLC.

## 2. Material and Methods

### 2.1. Patients and Samples

Our study group consisted of 53 patients (13 females and 40 males) aged between 47 to 75 years (average age: 63.4 ± 7.69 years) who were diagnosed with primary NSCLC and had undergone surgery in the Thoracic Surgery Ward of the Specialist Hospital of Lung Diseases and Tuberculosis in Bystra Slaska, Poland, between 2009 and 2010. Sociodemographic characteristics, such as age, sex, and smoking status (nonsmokers and active smokers), were collected using a standard questionnaire. Tumor and adjacent noncancerous tissues after excision were evaluated for clinical parameters, such as histopathological type (adenocarcinoma/squamous cell carcinoma), pathological staging of the tumor (pTNM), and the grade of differentiation (G), independently by two pathomorphologists.

### 2.2. Preparation of Tissues

Tumor and adjacent noncancerous lung parenchymatous tissues (taken at a distance of not less than 5 cm from the visible edge of the tumor) were obtained at the time of surgical resection. Each sample was placed in a separate tube, stored at -20 °C, and transported to Department of Medical and Molecular Biology in Zabrze, Poland for determining the concentration of MDA and activity of SOD, Cu/ZnSOD, MnSOD, CAT, GPx, GR, and GST. Tissue samples were cut into small pieces, homogenized in 0.9% NaCl on ice (0.3 g of tissue in 2.7 ml NaCl) in short cycles of a few seconds, and sonicated to disintegrate the cell membranes using a UP50H ultrasonic processor (Hielscher Ultrasonics GmbH, Germany). Tissue homogenates were centrifuged at 13,000 rpm for 10 minutes at 4 °C, and the supernatants were frozen at -80 °C until biochemical parameters were analyzed. The study protocol was approved by The Ethical Committee of the Medical University of Silesia in Katowice, Poland (KNW/0022/KB1/119/I/09). All the subjects were enrolled voluntarily after being informed about the scope and goal of this trial.

### 2.3. Biochemical Analyses

#### 2.3.1. Determination of MDA Concentration

MDA concentration was measured fluorometrically using thiobarbituric acid (TBA) according to the method of Ohkawa et al. [21]. The method was slightly modified by adding sodium sulfate and 3,5-diisobutyl-4-hydroxytoluene to increase the specificity of the reaction. Fluorescence was read at the excitation and emission wavelengths of 515 and 552 nm, respectively, on an LS 45 fluorescence spectrometer (PerkinElmer, USA). Concentration of MDA was calculated by using a standard curve prepared from 1,1,3,3-tetraethoxypropane. Data was expressed as *μ*moles MDA per 1 g of total protein (*μ*mol/g).

#### 2.3.2. Determination of SOD Activity

The activity of SOD (EC.1.15.1.1) in tissue homogenates was determined by following the method of Oyanagui [22]. A superoxide anion radical (O_2_^−^), produced in the reaction catalysed by xanthine oxidase, reacts with hydroxylamine to form nitric ion. Nitric ion combines with naphthalene diamine and sulfaniline acid producing a colored product. The concentration of this colored product is proportional to the activity of SOD in the samples. The absorbance was read at 560 nm on a Victor X3 Light Plate Reader (PerkinElmer, USA). Enzymatic activity was expressed as nitrite units (NU) per 1 mg of protein in tissue. One NU is defined as 50% inhibition of nitrite ion formation under the method's condition. KCN was used as the inhibitor of the Cu/ZnSOD isoenzyme. Cu/ZnSOD activity was calculated as the difference between total SOD activity and MnSOD activity.

#### 2.3.3. Determination of CAT Activity

CAT (EC.1.11.1.9) activity was measured in the supernatant of the lung homogenates by following the kinetic method of Aebi [23]. Briefly, 50 mM Tris/HCl buffer, pH 7.4, and perhydrol were mixed with 50 *μ*l of homogenate. After 10 seconds, the absorbance was read at 240 nm every 30 seconds for 2 minutes using a Shimadzu UV-1700 PharmaSpec UV-Vis Spectrophotometer (Kyoto, Japan). Enzymatic activity was expressed as International Unit (IU) per 1 g of total protein (IU/g of total protein).

#### 2.3.4. Determination of GPx Activity

GPx (E.C.1.11.1.9) activity was measured by following the method of Paglia and Valentine by using GSH and *tert*-butyl peroxide as substrates [24]. The kinetics of changes in absorbance were read at 355 nm on a PerkinElmer Victor X3 (PerkinElmer, USA). The activity of GPx was expressed as the quantity of *μ*moles of a reduced form of nicotinamide adenine dinucleotide phosphate (NADPH+H^+^) required to recover GSH in 1 minute, and expressed as IU/g of total protein.

#### 2.3.5. Determination of GST Activity

The activity of GST (*EC* 2.5.1.18) was measured according to the kinetic method described by Habig and Jakoby [25]. In this method, GST reacts with 1-chloro-2,3-dinitrobenzene producing a thioether. The change in absorbance at 355 nm was monitored using a PerkinElmer Victor X3 reader. One unit of GST was defined as micromoles of thioether produced in 1 minute. The results were expressed as IU/g protein.

#### 2.3.6. Determination of GR Activity

The activity of GR (E.C.1.6.4.2) was measured in the supernatant of tissue homogenates by following Richterich's kinetic method [26], where oxidized glutathione (GSSG) was used as a substrate. Changes in absorbance were read at 355 nm on a Victor X3 Light Plate Reader (PerkinElmer, USA). Enzyme activity was determined as *μ*moles of NADPH+H^+^ required to replenish the concentration of GSH in 1 minute, and expressed as IU/g protein.

#### 2.3.7. Protein Concentration

Protein concentration in the samples was determined by Lowry's method using bovine serum albumin as a standard [27].

### 2.4. Statistical Analysis

All statistical analyses were performed using Statistica 13.1 (StatSoft, USA). The normality of the result distribution was verified. Data is presented as mean value ± standard deviation (SD). To determine the statistical significance of differences among various experimental groups, *t*-test or Mann-Whitney's test was performed. The correlation between different variables was calculated using Pearson's linear correlation coefficient. Statistical significance was set at a *p* value ≤ 0.5. The lack of statistical significance is presented as NS (nonsignificant).

## 3. Results

The sociodemographic characteristics and clinical and pathological features of the study participants are presented in Table 1.

The concentrations of MDA and the activities of antioxidant enzymes in the tumor and adjacent noncancerous tissues from patients with NSCLC were compared. No significant differences were observed in the concentrations of MDA and activities of MnSOD and Cu/ZnSOD, while the activities of CAT, GPx, GR, and GST were found to be significantly altered in tumor tissues compared with those in the adjacent noncancerous tissues. The activities of SOD and CAT were significantly higher in the adjacent noncancerous tissues than those in tumor tissues, while the activities of GSH-related enzymes were significantly higher in tumor tissues than in the adjacent noncancerous tissues (Table 2).

The abovementioned parameters were then compared based on the histological types of tumor. We found that the activity of GSH-related enzymes was significantly higher in cancer tissues of both adenocarcinoma and squamous cell carcinoma than in the noncancerous tissues, while the activity of CAT was significantly higher in the adjacent noncancerous tissues than in the tumor tissues. The activities of SOD and MnSOD were found to be significantly high only in noncancerous tissues of patients with squamous cell carcinoma, while their activities did not change significantly in patients diagnosed with adenocarcinoma (Table 3).

No significant difference in the activity of antioxidant enzymes and concentration of MDA was observed between tumor and adjacent noncancerous tissues of patients with adenocarcinoma and squamous cell carcinoma (Table 4).

Similarly, no significant difference was observed in the concentrations of MDA and activities of antioxidant enzymes between the tumor and adjacent noncancerous tissues of patients with different differentiation grades of tumor (G1, G2, and G3), tumor size (T1, T2, and T3), and metastatic lymph nodes (N0, N1, and N2) (data not shown).

Furthermore, the correlation between the activity of antioxidant enzymes and age, and the sex of the patients was also examined. The results are presented in Table 5. The activity of GST was found to be significantly higher in tumor tissues of patients who were more than 65 years old than in the group of younger patients (*p* = 0.02). The activities of other antioxidant enzymes did not change significantly between the two groups.

We did not find any change in the activity of antioxidant enzymes between males and females, except for the activity of GPx, which was significantly higher in women than in men (*p* = 0.009).

We also analyzed the relationship between the activity of antioxidant enzymes in tumor tissues and factors associated with carcinogenicity, such as smoking, and COPD; however, no significant relationship was observed between these parameters (data not shown).

## 4. Discussion

In patients with lung cancer, only a 16% 5-year survival rate is noted worldwide. The number of deaths due to lung cancer is expected to rise to 10 million deaths per year by 2030 [1, 2, 29]. The factors that cause lung cancer are complex and not yet fully understood. In recent years, the levels of ROS have aroused interest as signal molecules required for regulating various biological processes. It has been shown that cancer cells have increased ROS levels in comparison to their normal counterparts. This is due to an altered metabolism and mitochondrial dysfunction in cancer cells [30, 31]. ROS play a dual role as both deleterious and beneficial molecules. The “two-faced” character of ROS results from the fact that ROS within cells act as secondary messengers in intracellular signaling cascades, which induce and maintain the oncogenic phenotype of cancer cells. However, ROS can also induce cellular senescence and apoptosis and can therefore function as an antitumorigenic factor [32].

### 4.1. MDA

MDA, formed under oxidative stress conditions, is an end-product generated by the decomposition of arachidonic acid and larger polyunsaturated fatty acids, and has the ability to react with biomolecules, such as proteins or DNA [33, 34]. It has been widely used as a biomarker for lipid peroxidation, and it is known to be associated with different pathological conditions; however, its biological activity has not been studied in a dose-dependent manner [11, 35]. Moreover, several studies have evaluated the concentration of MDA in serum or urine samples of patients; however, that does not reflect the extent of oxidative damage caused by ROS in a given tissue or organ. In this study, MDA concentrations were not found to be significantly different between tumor and adjacent normal tissues of patients with NSCLC (Table 2), irrespective of the difference in histological types of NSCLC (Tables 3 and 4) or the age and sex of the patients (Table 5). Gegotek et al. reported significantly high levels of reactive aldehydes in tumor tissues of patients with NSCLC than in noncancerous tissues [36]. In a group of Algerian lung cancer patients, concentrations of MDA, determined by using the TBA method similar to our study, were significantly high in tumor tissues than in the peritumoral stroma [37]. They were also several times higher than those measured in our study. However, the TBA reacting substances (TBARs) test is known for its nonspecificity, which has led to substantial controversy over its use for the quantification of MDA levels [11]. An adequate method for the determination of MDA levels in appropriate biological samples remains to be determined.

Antioxidant enzymes constitute the main defense mechanism of lung tissues against ROS-mediated injury, and their activity increases in response to oxidative stress. This has been shown to minimize ROS-mediated injury in experimental systems indicating that antioxidant levels might help in determining the role of ROS in the initiation of lung carcinogenesis [38]. In this study, we aim to evaluate the activity of antioxidant enzymes in the tumor and adjacent noncancerous tissues.

### 4.2. SOD

The first line of protection against ROS includes three isoforms of SOD, namely cytosolic Cu/ZnSOD, mitochondrial MnSOD, and extracellular SOD, which are present in the epithelial lining of blood vessels [38]. They play a major role in protecting the lungs against free radicals produced as a part of normal metabolism and also prevent the progression of oxidative stress-related lung diseases. In the present study, the activity of Cu/ZnSOD, MnSOD, and SOD was found to be higher in the adjacent noncancerous tissues than in the tumor tissues; however, this effect was significant only for SOD activity (*p* = 0.016). The results of this study are in contrast with those of Chung-man Ho et al. who demonstrated that the activity of SOD was significantly higher in the tumor tissues than in the adjacent tumor-free lung tissues in patients with NSCLC (*p* = 0.035) [38]. When the activities of antioxidant enzymes were compared according to the histological types of NSCLC, we observed that the activities of SOD and MnSOD in the adjacent noncancerous tissues were significantly higher in patients with squamous cell carcinoma (Table 3) than in patients with adenocarcinoma. The results obtained by Svensk et al. suggested that the activity of MnSOD increased in lung carcinoma, and this increase was more prominent in patients with squamous cell carcinoma than in patients with the other types of lung carcinomas [14]. Interestingly, no significant difference was observed in the activity of different isoforms of SOD between the adjacent nonmalignant tissues of adenocarcinoma and squamous cell carcinoma (Table 4). As rightly discussed by Kinnula and Crapo [5], it is difficult to compare the activities of the three different isoforms of SOD in the lung because they are located in compartments of different sizes and have been evaluated by several investigators using different assays, each having different sensitivities and specificities.

The association of aging with oxidative stress is undisputable. Aging, resulting from the accumulation of molecular damages in DNA, proteins, and lipids, is characterized by an increase in the intracellular levels of oxidative stress. Therefore, in this study, we compared the activity of antioxidant enzymes in tumor tissues of patients with NSCLC by categorizing them in two age groups—below and above 65 years of age [39]. The activity of the three different isoforms of SOD did not change significantly between the tumor and noncancerous tissues of patients above or below 65 years of age. Similarly, no significant difference was observed in the activity of SOD between females and males (Table 5).

### 4.3. CAT

CAT, located mainly in peroxisomes, decomposes H_2_O_2_, a by-product of fatty acid oxidation, to oxygen and water. Thus, CAT confers protection against the toxic effects of H_2_O_2_ without generating intermediate free radicals, and the resulting oxygen is utilized for other metabolic processes [40]. A significant reduction in the activity of CAT has been observed in many types of cancer: head and neck, lungs, gastrointestinal tract, breasts, kidney, or leukemia [41]. In this study, CAT activity in NSCLC patients was significantly high (*p* = 0.00001) in the adjacent noncancerous tissues (Table 2), irrespective of the different histological types of lung cancer (Table 3). Similar results were also obtained by Otsmane et al. [37]. Ho et al. [38] proposed that inflammation in the lungs may contribute to the decreased activity of catalase, resulting in an increased concentration of intracellular H_2_O_2_ and the promotion of cancer.

### 4.4. GSH-Related Enzymes

The levels of GSH and enzymes required for maintaining its levels in turn have been suggested to constitute one of the basic antioxidant defense mechanisms of the human lungs [17].

Previous studies suggested that the antioxidant activity is impaired in lung cancers, and the expression of GSH-related antioxidant enzymes creates an interindividual risk factor for lung cancer [42]. In the present study, the activity of GSH-related antioxidant enzymes, namely, GPx, GST, and GR, were significantly higher in the tumor tissues than in the adjacent noncancerous lung tissues (Table 2) in patients with adenocarcinoma and squamous cell carcinoma (Table 3). However, the activity of these enzymes was not found to be significantly different between the two histological types of NSCLC (Table 4). Since GSH is essential for mounting successful immune response by activating T-lymphocytes and polymorphonuclear leukocytes for producing cytokines [43], there could be a link between the activity of GSH-related enzymes and tumor biology. Cancer tissue might be superior to adjacent noncancerous tissue in terms of its ability to decrease oxidative stress, thereby, facilitating tumor growth. The mediators and cellular effectors of inflammation are important constituents of the local environment of tumor tissue. Thus, cancer cells are able to protect themselves by increasing the intracellular concentrations of GSH. Inflammation in the tumor microenvironment aids in the proliferation and survival of malignant cells; promotes angiogenesis and metastasis; and alters immune response, response to hormones, and chemotherapeutic agents [44, 45]. Contrary to previously analyzed enzymes determined in our study, only GSH-related molecules have shown differences according to age: surprisingly GST activity was higher in the tumor tissue of patients above 65 years of age (*p* = 0.02, Table 5). Interestingly, females with NSCLC exhibited a significantly higher activity of GPx in tumor tissues relative to men.

The link between inflammation and cancer has also been confirmed by anti-inflammatory therapies that have proven to be effective in the prevention and treatment of cancer [46]. Therefore, the relationship between the activity of antioxidant enzymes and COPD was examined. Damage to the lungs in COPD is caused by oxidative stress (both exogenous resulting from smoking and endogenous), release of inflammatory cytokines, protease activity (due to the imbalance in protease : antiprotease ratio), and expression of autoantibodies [47]. This in turn can lead to airway destruction, air trapping, and lung hyperinflation [48]. Smoking is believed to be the primary cause of lung cancer. The adverse action of cigarette smoke is due to the presence of a large variety of compounds like nicotine, benzo(a)pyrene, oxidants, and free radicals that initiate, promote, and/or amplify oxidative damage [49]. Several studies have reported that cigarette smoking is associated with an increase in the incidence and severity of various diseases, such as lung cancer and COPD [6, 50]. COPD and lung cancer are caused by cigarette smoking, and there is increasing evidence linking the two diseases beyond just a common etiology. COPD is an independent risk factor for lung carcinoma, particularly for squamous cell carcinoma, and lung cancer is up to five times more likely to occur in smokers with airflow obstruction than those with normal lung function [51]. Villeneuve et al. reported that the lifetime risk of developing lung cancer is 17.2% and 11.6% for males and females, respectively, among smokers, when compared with 1.3% for males and 1.4% for females among nonsmokers [52]. In this study, we evaluated the activity of antioxidant enzymes in the tumor and adjacent noncancerous tissues obtained from patients with NSCLC. We did not find any significant difference in the activity of antioxidant enzymes between smokers and nonsmokers, and patients with or without COPD. It is possible that smoking or other inflammatory lung diseases do not have a pivotal impact on oxidative stress nascency in the initial stage of cancer development.

The incidence rate of lung cancer is declining in men and plateauing in women after increasing for several decades. This lag in the temporal trend of lung cancer incidence rates in women compared with that in men is reflected in historical differences in cigarette smoking habits between men and women; cigarette smoking in women peaked about 20 years later than in men [1]. Our results suggested that the age and sex of the patients do not have any effect on the activity of antioxidant enzymes in the tumor tissues of NSCLC patients, except for the activity of GPx and GST as mentioned in Table 5. The activity of antioxidant enzymes was not found to be correlated with differentiation grade, tumor size, and metastatic lymph nodes (data not shown).

In majority of the studies, the activity of antioxidant enzymes has been determined in the peripheral blood erythrocytes of patients with neoplastic diseases and not in the cancerous tissues. In this study, we evaluated the activities of antioxidant enzymes in the homogenates of lung tissue, and therefore, our results cannot always be directly compared with those reported by others. Moreover, the percentage of adeno- and squamous cell carcinomas in patients recruited in this study was different from the statistics available for general lung cancer: 32% and 67% of the cases of adenocarcinoma and squamous cell carcinoma, respectively, in this study versus 40% and 30% of the cases according to available statistics. Therefore, we have discussed our results only for NSCLC, in general, and compared the activity of antioxidant enzymes between the two histological types of lung cancer. Further studies with a large cohort of patients with NSCLC are warranted to conclusively prove our observations.

## 5. Conclusion

A significant change in the activity of antioxidant enzymes was observed during the process of carcinogenesis. Tumor cells always had low MnSOD activity, usually low Cu/ZnSOD activity, and almost always low catalase activity compared with those of the corresponding normal tissues. Activities of GSH-related enzymes were significantly high in lung cancer tissues, irrespective of the histological type of lung cancer, which could possibly be the way by which tumor cells protect themselves against increased oxidative stress.

## Figures and Tables

**Table 1:** The clinical and pathological features of NSCLC patients.

Variables		Number of patients (%) 53 (100)
Sex	F	13 (24.53)
	M	40 (75.47)

Age	≤65 years	31 (58.49)
	>65 years	22 (41.51)

Histology	Squamous cell carcinoma	36 (67.93)
	Adenocarcinoma	17 (32.07)

Differentiation grade	G1	0 (0)
	G2	21 (39.62)
	G3	32 (60.38)

T factor	T1	12 (22.64
	T2	34 (64.15)
	T3	7 (13.21)

N factor	N0	27 (50.94)
	N1	18 (33.96)
	N2	8 (15.10)

COPD	Yes	22 (41.51)
	No	31 (58.49)

Smoking status	Smokers	39 (73.58)
	Nonsmokers	13 (24.53)
	No data	1 (1.89)

Abbreviations: G—histopathological cancer grading (G1: well differentiated; G2: moderately differentiated; G3: poorly differentiated; G4: undifferentiated); T—tumor size (T1: size ≤ 3 cm; T2: size > 3 cm to ≤5 cm; T3: size > 5 cm to 7 cm); N—metastatic lymph nodes (N0: no regional lymph node metastasis; N1: metastasis in ipsilateral peribronchial and/or hilar lymph node and intrapulmonary node; N2: metastasis in ipsilateral mediastinal and/or subcarinal lymph nodes); COPD—chronic obstructive pulmonary disease [28].

**Table 2:** Concentrations of MDA and activities of antioxidant enzymes in the tumor and adjacent noncancerous tissues of patients with NSCLC.

Parameters	Tumor tissue	Adjacent noncancerous tissue	*p* value
MDA (*μ*mol/g)	0.69 ± 0.78	0.47 ± 0.14	NS
SOD (NU/mg)	16.26 ± 3.96	18.53 ± 4.51	**0.016**
MnSOD (NU/mg)	9.91 ± 4.55	11.28 ± 3.85	NS
Cu/ZnSOD (NU/mg)	6.37 ± 4.26	7.25 ± 2.07	NS
CAT (IU/g)	49.79 ± 36.99	130 ± 49.97	**0.00001**
GPx (IU/g)	8.61 ± 6.34	5.58 ± 2.66	**0.004**
GST (IU/g)	3.82 ± 3.46	1.61 ± 1.09	**0.00024**
GR (IU/g)	27.27 ± 22.85	9.86 ± 5.21	**<0.001**

NS—not significant.

**Table 3:** Concentrations of MDA and activities of antioxidant enzymes in the cancerous and noncancerous tissues of patients with adenocarcinoma and squamous cell carcinoma.

Parameters	Adenocarcinoma			Squamous cell carcinoma		
	Tumor	Adjacent noncancerous tissue	*p* value	Tumor	Adjacent noncancerous tissue	*p* value
MDA (*μ*mol/g)	0.57 ± 0.44	0.47 ± 0.18	NS	0.74 ± 0.93	0.47 ± 0.13	NS
SOD (NU/mg)	16.88 ± 3.77	17.28 ± 5.31	NS	15.98 ± 4.08	19.15 ± 4.02	**0.008**
MnSOD (NU/mg)	11.2 ± 4.0	10.4 ± 4.31	NS	9.34 ± 4.53	11.7 ± 3.61	**0.04**
Cu/ZnSOD (NU/mg)	5.76 ± 4.29	6.86 ± 2.6	NS	6.69 ± 4.28	7.44 ± 1.78	NS
CAT (IU/g)	45.4 ± 24.93	130.64 ± 50.2	**<0.001**	51.85 ± 41.84	130.42 ± 50.7	**<0.001**
GPx (IU/g)	10.75 ± 7.54	5.95 ± 2.47	**0.04**	7.58 ± 5.52	5.4 ± 2.73	**0.05**
GST (IU/g)	3.14 ± 2.16	1.5 ± 1.3	**0.04**	4.14 ± 3.91	1.60 ± 0.95	**0.002**
GR (IU/g)	28.43 ± 16.36	8.94 ± 3.39	**<0.001**	26.88 ± 25.51	10.31 ± 5.89	**0.002**

NS—not significant.

**Table 4:** Concentration of MDA and activity of antioxidant enzymes in tumor and adjacent noncancerous tissues of patients with adenocarcinoma and squamous cell carcinoma.

Parameters	Tumor			Adjacent noncancerous tissue		
	Adenocarcinoma	Squamous cell carcinoma	*p* value	Adenocarcinoma	Squamous cell carcinoma	*p* value
MDA (*μ*mol/g)	0.91 ± 1.30	0.94 ± 1.27	NS	0.47 ± 0.18	0.48 ± 0.11	NS
SOD (NU/mg)	16.14 ± 4.24	16.45 ± 4.04	NS	17.28 ± 5.31	19.15 ± 4.02	NS
MnSOD (NU/mg)	10.74 ± 4.25	10.25 ± 5.00	NS	10.40 ± 4.31	11.70 ± 3.60	NS
Cu/ZnSOD (NU/mg)	5.45 ± 3.94	6.20 ± 4.15	NS	6.86 ± 2.60	7.44 ± 1.80	NS
CAT IU/g)	61.51 ± 62.22	48.78 ± 39.90	NS	130.64 ± 50.30	130.42 ± 50.71	NS
GPx (IU/g)	10.31 ± 7.02	7.03 ± 5.93	NS	5.94 ± 2.47	5.40 ± 2.73	NS
GST (IU/g)	2.90 ± 2.11	4.15 ± 3.74	NS	1.60 ± 1.30	1.60 ± 0.95	NS
GR (IU/g)	26.65 ± 16.46	28.22 ± 25.00	NS	8.94 ± 3.39	10.31 ± 5.89	NS

NS—not significant.

**Table 5:** Effect of age and sex of patients on the activity of antioxidant enzymes and concentration of MDA in tumor tissues.

Parameters	Age			Sex		
	≤65 years	>65 years	*p* value	Female	Male	*p* value
MDA (*μ*mol/g)	0.81 ± 1.27	1.14 ± 1.27	NS	0.59 ± 0.28	1.04 ± 1.45	NS
SOD (NU/mg)	16.08 ± 4.6	16.76 ± 3.22	NS	16.52 ± 4.05	16.30 ± 4.10	NS
MnSOD (NU/mg)	10.91 ± 4.65	9.71 ± 4.87	NS	11.62 ± 3.87	10.01 ± 4.97	NS
Cu/ZnSOD (NU/mg)	5.19 ± 3.49	7.04 ± 4.61	NS	5.01 ± 4.38	6.28 ± 3.96	NS
CAT (IU/g)	59.71 ± 51.32	43.23 ± 41.98	NS	48.72 ± 31.97	54.21 ± 52.39	NS
GPx (IU/g)	7.93 ± 6.33	8.31 ± 6.67	NS	12.02 ± 6.16	6.81 ± 6.02	**0.009**
GST (IU/g)	2.85 ± 2.46	5.01 ± 3.99	**0.02**	3.00 ± 1.82	4.00 ± 3.68	NS
GR (IU/g)	23.45 ± 15.52	33.71 ± 29.00	NS	24.72 ± 17.77	28.69 ± 23.92	NS

NS—not significant.

## Data Availability

The data used to support the findings of this study are available from the corresponding author upon request.

## References

[1] Jemal A., Siegel R., Ward E. (2008). Cancer Statistics, 2008. *CA: A Cancer Journal for Clinicians*.

[2] McErlean A., Ginsberg M. S. (2011). Epidemiology of lung cancer. *Seminars in Roentgenology*.

[3] Herbst R. S., Heymach J. V., Lippman S. M. (2008). *New England Journal of Medicine*.

[4] Reck M. (2015). Management of non-small-cell lung cancer: recent developments. *The Lancet*.

[5] Kinnula V. L., Crapo J. D. (2003). Superoxide dismutases in the lung and human lung diseases. *American Journal of Respiratory and Critical Care Medicine*.

[6] Kirkham P. A., Barnes P. J. (2013). Oxidative stress in COPD. *Chest*.

[7] Murphy M. P. (2009). How mitochondria produce reactive oxygen species. *The Biochemical Journal*.

[8] Reuter S., Gupta S. C., Chaturvedi M. M., Aggarwal B. B. (2010). Oxidative stress, inflammation, and cancer: how are they linked?. *Free Radical Biology & Medicine*.

[9] Nicco C., Laurent A., Chereau C., Weill B., Batteux F. (2005). Differential modulation of normal and tumor cell proliferation by reactive oxygen species. *Biomedicine & Pharmacotherapy*.

[10] Lymperaki E., Makedou K., Iliadis S., Vagdatli E. (2015). Effects of acute cigarette smoking on total blood count and markers of oxidative stress in active and passive smokers. *Hippokratia*.

[11] Ayala A., Muñoz M. F., Argüelles S. (2014). Lipid peroxidation: production, metabolism, and signaling mechanisms of malondialdehyde and 4-hydroxy-2-nonenal. *Oxidative Medicine and Cellular Longevity*.

[12] Fukai T., Ushio-Fukai M. (2011). Superoxide Dismutases: Role in Redox Signaling, Vascular Function, and Diseases. *Antioxidants & Redox Signaling*.

[13] De Palma G., Mozzoni P., Acampa O. (2010). Expression levels of some antioxidant and epidermal growth factor receptor genes in patients with early-stage non-small cell lung cancer. *Journal of Nucleic Acids*.

[14] Svensk A.-M., Soini Y., Pääkkö P., Hirvikoski P., Kinnula V. L. (2004). Differential expression of superoxide dismutases in lung cancer. *American Journal of Clinical Pathology*.

[15] Wu G., Fang Y. Z., Yang S., Lupton J. R., Turner N. D. (2004). Glutathione metabolism and its implications for health. *The Journal of Nutrition*.

[16] Cantin A. M., North S. L., Hubbard R. C., Crystal R. G. (1987). Normal alveolar epithelial lining fluid contains high levels of glutathione. *Journal of Applied Physiology*.

[17] Cheng S.-B., Liu H.-T., Chen S.-Y., Lin P.-T., Lai C.-Y., Huang Y.-C. (2017). Changes of oxidative stress, glutathione, and its dependent antioxidant enzyme activities in patients with hepatocellular carcinoma before and after tumor resection. *PLoS One*.

[18] Hayes J. D., McLellan L. I. (1999). Glutathione and glutathione-dependent enzymes represent a co-ordinately regulated defence against oxidative stress. *Free Radical Research*.

[19] Hayes J. D., Pulford D. J. (1995). The Glut athione S-transferase supergene family: regulation of GST and the contribution of the lsoenzymes to cancer chemoprotection and drug resistance Part I. *Critical Reviews in Biochemistry and Molecular Biology*.

[20] Sen C. K. (2000). Cellular thiols and redox-regulated signal transduction. *Current Topics in Cellular Regulation*.

[21] Ohkawa H., Ohishi N., Yagi K. (1979). Assay for lipid peroxides in animal tissues by thiobarbituric acid reaction. *Analytical Biochemistry*.

[22] Oyanagui Y. (1984). Reevaluation of assay methods and establishment of kit for superoxide dismutase activity. *Analytical Biochemistry*.

[23] Aebi H. (1984). [13] Catalase _in vitro_. *Methods in Enzymology*.

[24] Paglia D. E., Valentine W. N. (1967). Studies on the quantitative and qualitative characterization of erythrocyte glutathione peroxidase. *The Journal of Laboratory and Clinical Medicine*.

[25] Habig W. H., Jakoby W. B. (1981). [51] Assays for differentiation of glutathione S-Transferases. *Methods in Enzymology*.

[26] Richterich R. (1969). *Clinical chemistry: theory and practice*.

[27] Lowry O. H., Rosebrough N. J., Farr A. L., Randall R. J. (1951). Protein measurement with the Folin phenol reagent. *The Journal of Biological Chemistry*.

[28] Lim W., Ridge C. A., Nicholson A. G., Mirsadraee S. (2018). The 8th lung cancer TNM classification and clinical staging system: review of the changes and clinical implications. *Quantitative Imaging in Medicine and Surgery*.

[29] Jemal A. (2011). Global cancer statistics. *CA: A Cancer Journal for Clinicians*.

[30] Galadari S., Rahman A., Pallichankandy S., Thayyullathil F. (2017). Reactive oxygen species and cancer paradox: to promote or to suppress?. *Free Radical Biology and Medicine*.

[31] Aykin-Burns N., Ahmad I. M., Zhu Y., Oberley L. W., Spitz D. R. (2009). Increased levels of superoxide and H2O2mediate the differential susceptibility of cancer cells versus normal cells to glucose deprivation. *The Biochemical Journal*.

[32] Valko M., Rhodes C. J., Moncol J., Izakovic M., Mazu M. (2006). Free radicals, metals and antioxidants in oxidative stress-induced cancer. *Chemico-Biological Interactions*.

[33] Esterbauer H., Schaur R. J., Zollner H. (1991). Chemistry and biochemistry of 4-hydroxynonenal, malonaldehyde and related aldehydes. *Free Radical Biology & Medicine*.

[34] Niedernhofer L. J., Daniels J. S., Rouzer C. A., Greene R. E., Marnett L. J. (2003). Malondialdehyde, a product of lipid peroxidation, is mutagenic in human cells. *The Journal of Biological Chemistry*.

[35] Garcia S. C., Grotto D., Bulcão R. P. (2013). Evaluation of lipid damage related to pathological and physiological conditions. *Drug and Chemical Toxicology*.

[36] Gegotek A., Niklinski J., Charkiewicz R., Bielawska K., Kozlowski M., Skrzydlewska E. (2014). Relationships between level of lipid peroxidation products and expression of Nrf2 and its activators/ inhibitors in non-small cell lung cancer tissue. *Free Radical Biology and Medicine*.

[37] Otsmane A., Kacimi G., Adane S., Cherbal F., Aouichat B. S. (2018). Clinico-epidemiological profile and redox imbalance of lung cancer patients in Algeria. *Journal of Medicine and Life*.

[38] Ho J. C., Zheng S., Comhair S. A., Farver C., Erzurum S. C. (2001). Differential expression of manganese superoxide dismutase and catalase in lung cancer. *Cancer Research*.

[39] Minelli A., Bellezza I., Conte C., Culig Z. (2009). Oxidative stress-related aging: a role for prostate cancer?. *Biochimica et Biophysica Acta*.

[40] Kirkman H. N., Gaetani G. F. (2007). Mammalian catalase: a venerable enzyme with new mysteries. *Trends in Biochemical Sciences*.

[41] Ścibior-Bentkowska D., Czeczot H. (2006). Katalaza*—*budowa, właściwości, funkcje. *Postȩpy Higieny i Medycyny Doświadczalnej*.

[42] Crawford E. L., Khuder S. A., Durham S. J. (2000). Normal bronchial epithelial cell expression of glutathione transferase P1, glutathione transferase M3, and glutathione peroxidase is low in subjects with bronchogenic carcinoma. *Cancer Research*.

[43] Townsend D. M., Tew K. D., Tapiero H. (2003). The importance of glutathione in human disease. *Biomedicine & Pharmacotherapy*.

[44] Mantovani A., Allavena P., Sica A., Balkwill F. (2008). Cancer-related inflammation. *Nature*.

[45] Grivennikov S. I., Greten F. R., Karin M. (2010). Immunity, inflammation, and cancer. *Cell*.

[46] Gonda T. A., Tu S., Wang T. C. (2009). Chronic inflammation, the tumor microenvironment and carcinogenesis. *Cell Cycle*.

[47] Brusselle G. G., Joos G. F., Bracke K. R. (2015). New insights into the immunology of chronic obstructive pulmonary disease. *Lancet*.

[48] Durham A. L., Adcock I. M. (2015). The relationship between COPD and lung cancer. *Lung Cancer*.

[49] Koul A., Bhatia V., Bansal M. (2001). Effect of alpha-tocopherol on pulmonary antioxidant defence system and lipid peroxidation in cigarette smoke inhaling mice. *BMC Biochemistry*.

[50] MacNee W. (2005). Treatment of stable COPD: antioxidants. *European Respiratory Review*.

[51] Young R. P., Hopkins R. J. (2010). Link between COPD and lung cancer. *Respiratory Medicine*.

[52] Villeneuve P., Mao Y. (1994). Lifetime probability of developing lung cancer, by smoking status Canada. *Canadian Journal of Public Health*.

